# Beyond mean heart dose: differentiated electrocardiographic manifestations and dosimetric mechanisms of cardiac injury in left-sided versus right-sided breast cancer after intensity-modulated radiotherapy

**DOI:** 10.3389/fonc.2026.1901038

**Published:** 2026-09-16

**Authors:** Yanjun Zhang, Xiaochen Han, Feifei Wang, Ruoling Han, Yasi Zhang, Ziqi Zhao, Dan Liu, Zifeng Chi

**Affiliations:** Department of Radiation Oncology, Fourth Hospital of Hebei Medical University, Shijiazhuang, China

**Keywords:** breast cancer, cardiac substructures, intensity-modulated radiotherapy (IMRT), laterality heterogeneity, sinoatrial node (SAN)

## Abstract

**Objective:**

To investigate dosimetric exposure variations to the whole heart and substructures between left- and right-sided breast cancer patients receiving intensity-modulated radiotherapy (IMRT), and to evaluate their relationships with post-radiotherapy (post-RT) electrocardiogram (ECG) alterations.

**Methods:**

Clinical and dosimetric data of 91 breast cancer patients (54 left-sided, 37 right-sided) undergoing postoperative free-breathing IMRT were retrospectively analyzed. Twelve-lead ECGs were regularly monitored during follow-up. Kaplan-Meier analysis evaluated ECG abnormality-free survival. Receiver operating characteristic (ROC) curves compared the predictive values of mean heart dose (MHD) and left ventricular mean dose (LV *D*_mean_) for new-onset repolarization abnormalities in left-sided patients, utilizing mathematically derived population-specific split-points.

**Results:**

Target volume configurations (comprehensive regional and internal mammary node irradiation) drove distinct substructure exposure patterns between sides. In left-sided patients, MHD, LV, and left anterior descending artery (LAD) doses were significantly higher than those in right-sided patients (all *P* < 0.001). Conversely, right-sided patients sustained significantly higher doses to the sinoatrial node (SAN) and right coronary artery (RCA) (all *P* < 0.001). Left-sided patients had a significantly shorter median ECG abnormality-free survival than right-sided patients (7.0 vs. 12.0 months, *P* = 0.038). Among the 60 patients developing post-RT ECG modifications (incidence: 81.5% in left-sided cases), left-sided cases predominantly exhibited repolarization abnormalities (68.2%), whereas right-sided cases primarily presented rhythm abnormalities (62.5%, *P* = 0.032). In the left-sided subgroup, LV dosimetric parameters (LV *D*_mean_, LV *V*_5_–*V*_80_) were significantly higher in the repolarization group than in the rhythm group (all *P* < 0.01). ROC analysis indicated that LV *D*_mean_ outperformed MHD in predicting early repolarization changes (AUC: 0.776 vs. 0.715).

**Conclusion:**

Early post-RT cardiotoxicity follows a laterality-specific “dual-mechanism model.” Left-sided modifications manifest primarily as acute/subacute repolarization surrogates driven by diffuse LV exposure, whereas right-sided modifications present as rhythm-based stress from focal SAN exposure.

## 1 Introduction

Radiotherapy is crucial in breast cancer management (1), yet radiation-induced heart disease (RIHD) remains a prominent toxicity compromising long-term survival (2, 3). Landmark studies established a linear relationship between major coronary events and whole-heart dose (4). Nevertheless, recent evidence emphasizes that subclinical cardiotoxicity, manifesting as early, acute/subacute electrophysiological alterations and localized microvascular stress, serves as an occult, potentially transient precursor to clinical cardiac events (5–7).

Modern intensity-modulated radiotherapy (IMRT) enhances target conformality but expands the low-dose bath in surrounding organs. Crucially, the heart contains distinct, radiosensitive substructures (8, 9). Traditional mean heart dose (MHD) averages the entire cardiac volume, failing to capture localized high-dose spots within critical sub-volumes, which limits its ability to predict specific cardiotoxicity phenotypes (10, 11).

Because the heart is anatomically levorotated, cardiac spatial exposure diverges fundamentally between left- and right-sided breast fields (12, 13). This spatial discrepancy is further pronounced in high-risk cases requiring comprehensive regional nodal irradiation (RNI), particularly encompassing the internal mammary node (IMN) drainage area. While historical research focused heavily on left ventricular (LV) and left anterior descending (LAD) coronary artery exposure during left-sided treatment, the early transient electrophysiological impact on the right-sided conduction system—specifically the sinoatrial node (SAN) during right-sided treatment—remains poorly understood.

Therefore, this retrospective study compares whole-heart and substructure dosimetry under free-breathing IMRT between left- and right-sided breast cancer patients enrolled within a specific historical timeframe. Furthermore, we analyze post-radiotherapy ECG alterations to validate laterality specificity and identify population-specific statistical cut-offs descriptive of these early physiological surrogates via receiver operating characteristic (ROC) analysis.

## 2 Materials and methods

### 2.1 Patient selection

Clinical data of 91 female patients with unilateral breast cancer who underwent postoperative adjuvant IMRT between October 2023 and May 2024 were retrospectively reviewed. Inclusion criteria required histopathologically confirmed unilateral primary breast cancer managed with breast-conserving surgery or modified radical mastectomy, and normal baseline 12-lead ECG and echocardiography. Exclusion criteria comprised pre-existing severe cardiovascular diseases, prior thoracic radiotherapy, cardiac pacemakers, or non-radiotherapy-related cardiovascular events during follow-up.

The cohort included 54 left-sided and 37 right-sided patients. No statistically significant differences existed between the two groups regarding baseline clinical characteristics (all *P* > 0.05), (**Supplementary Table 1**). This study was formally approved by the Institutional Ethics Committee of The Fourth Hospital of Hebei Medical University (Approval No. 2022KY221). To eliminate confounding effects, the acute toxic periods of chemotherapy were strictly excluded during post-RT ECG assessment.

### 2.2 Radiotherapy and treatment planning

CT simulation and postoperative IMRT were performed under free-breathing conditions, delivering 50.0–50.4 Gy in 25 fractions to the PTV. This high-risk cohort required extensive regional treatment: 62.96% (34/54) of left-sided and 70.27% (26/37) of right-sided patients underwent mandatory internal mammary node irradiation (IMNI), with synchronized tumor bed or nodal boosts up to 60.0–60.2 Gy. Comprehensive target configurations and boost specifications are presented in **Supplementary Table 2** and **Supplementary Figure 1**.

All IMRT plans prioritized target coverage ≥ 95%. For complex left-sided IMN targets, relaxed compromise thresholds (whole heart *D*_mean_ < 8–10 Gy), whole heart *V*_30_ < 10%, and LAD *D*_max_ < 55Gy) were accepted under senior clinical discretion. Consequently, all plans were reviewed and officially approved by the Chief Radiation Oncologist to balance therapeutic coverage and organ protection.

### 2.3 Precise contouring of cardiac substructures and parameter extraction

Contouring of the whole heart, left ventricle (LV), right ventricle (RV), LAD, and right coronary artery (RCA) on planning CT scans was reviewed based on expert consensus guidelines (14, 15). Geometric proxy contouring methods were adopted for the sinoatrial node (SAN) and atrioventricular node (AVN), defining them as spheres with a 1.0-cm radius centered on the lateral wall of the superior vena cava-right atrium junction and the inferior interatrial septum (the Triangle of Koch), respectively (14, 15). All contours were cross-checked and approved by senior radiation oncologists. The mean dose (*D*_mean_), maximum dose (*D*_max_), and volume parameters *V*_5_, *V*_10_, V_20_, *V*_30_, and *V*_40_ were automatically extracted from the DVH.

### 2.4 ECG follow-up and criteria for electrophysiological abnormalities

Standard 12-lead ECGs were monitored before RT and during follow-up (months 3, 6, and 12, or upon symptom onset). Post-RT ECG abnormalities were defined as new-onset alterations compared with baseline, strictly framed as acute/subacute physiological surrogates of radiation-induced cardiac stress. These alterations were categorized into: (1) Rhythm abnormality: new-onset sinus tachycardia (>100 bpm), sinus bradycardia (<50 bpm), prominent sinus arrhythmia, frequent premature beats, new-onset atrial fibrillation, or conduction blocks; (2) Repolarization abnormality: non-specific ST-T changes (ST depression ≥0.05 mV; T-wave flattening/biphasic/inversion) and non-pharmacological QTc prolongation (>470 ms). The primary endpoint was the time to the first documentation of any new-onset ECG modification.

### 2.5 Statistical analysis

Statistical analyses were performed using SPSS version 27.0 and R software (version 4.3.0). Non-normally distributed metric data (Shapiro-Wilk test) are presented as Median (IQR) and compared using the Mann-Whitney U test, while categorical variables were analyzed via the chi-square or Fisher’s exact test. Abnormality cumulative incidence and event-free survival curves were estimated using the Kaplan-Meier method with the log-rank test. ROC curves evaluated dosimetric performance in predicting repolarization abnormalities. The cutoff values were rigorously determined using maximal selected rank statistics via the “maxstat” package in R software. A two-sided P < 0.05 denoted statistical significance.

## 3 Results

### 3.1 Reciprocal anatomical distribution patterns of radiation dose

Driven by spatial anatomy and target configurations, physical radiation exposure to cardiac substructures between left- and right-sided breast cancer radiotherapy exhibited completely reciprocal distribution patterns (**Table 1**).

**Table 1:** Comparison of dosimetric parameters between left-sided and right-sided breast cancer patients.

Parameter	Left-sided (n=54)	Right-sided (n=37)	*P* value
Whole Heart			
MHD(Gy)	7.78 (6.80, 9.20)	5.17 (4.52, 5.79)	<0.001
Heart *D*_max_ (Gy)	52.64 (49.88, 54.60)	29.33 (19.80, 35.74)	<0.001
Heart *V*_5_ (%)	37.07 (31.65, 44.58)	37.67 (29.02, 41.59)	0.39
Heart *V*_10_ (%)	19.61 (14.36, 23.23)	11.11 (7.10, 16.75)	<0.001
Heart *V*_20_ (%)	10.69 (8.15, 13.85)	0.61 (0.00, 2.49)	<0.001
Heart *V*_30_ (%)	5.84 (3.99, 7.66)	0.00 (0.00, 0.07)	<0.001
Sinoatrial node (SAN)			
SAN *D*_mean_ (Gy)	3.13 (2.47, 4.31)	8.22 (6.69, 12.70)	<0.001
SAN *D*_max_ (Gy)	4.85 (3.80, 6.02)	13.16 (10.40, 19.17)	<0.001
SAN *V*_5_ (%)	0.00 (0.00, 12.67)	100.00 (82.49, 100.00)	<0.001
SAN *V*_10_ (%)	0.00	13.42 (0.48, 85.84)	-†
Atrioventricular node (AVN)			
AVN *D*_mean_ (Gy)	2.92 (2.33, 4.35)	4.08 (2.50, 4.95)	0.075
AVN *D*_max_ (Gy)	4.30 (3.16, 6.46)	6.51 (4.16, 8.55)	0.009
AVN *V*_5_ (%)	0.00 (0.00, 12.24)	19.27 (0.00, 42.66)	0.012
Left ventricle (LV)			
LV *D*_mean_ (Gy)	9.49 (7.42, 10.57)	2.15 (1.75, 2.73)	<0.001
LV *D*_max_ (Gy)	50.39 (46.50, 53.19)	7.48 (5.82, 10.29)	<0.001
LV *V*_5_ (%)	37.96 (31.97, 47.80)	2.14 (0.25, 10.68)	<0.001
LV *V*_10_ (%)	23.79 (19.39, 28.39)	0.00 (0.00, 0.01)	<0.001
LV *V*_20_ (%)	15.88 (11.27, 19.07)	0.00	-†
LV *V*_30_ (%)	8.89 (5.25, 12.04)	0.00	-†
LV *V_4_*_0_ (%)	2.71 (1.02, 5.27)	0.00	-†
Right ventricle (RV)			
RV *D*_mean_ (Gy)	9.79 (7.46, 11.70)	5.12 (4.12, 6.10)	<0.001
RV *D*_max_ (Gy)	47.90 (43.20, 51.17)	16.65 (13.56, 22.76)	<0.001
RV *V*_5_ (%)	52.70 (44.27, 62.12)	39.36 (26.39, 46.32)	<0.001
RV *V*_10_ (%)	28.43 (18.77, 34.77)	6.07 (1.18, 11.08)	<0.001
RV *V*_20_ (%)	14.88 (7.36, 20.91)	0.00 (0.00, 0.15)	<0.001
RV *V*_30_ (%)	7.62 (2.29, 12.45)	0.00	-†
RV *V_4_*_0_ (%)	1.61 (0.10, 4.01)	0.00	-†
Left anterior descending artery (LAD)			
LAD *D*_mean_ (Gy)	39.38 (34.58, 43.89)	3.11 (2.27, 4.70)	<0.001
LAD *D*_max_ (Gy)	51.35 (49.09, 53.21)	5.91 (4.30, 7.11)	<0.001
LAD *V*_5_ (%)	100.00 (97.73, 100.00)	5.41 (0.00, 30.26)	<0.001
LAD *V*_10_ (%)	99.64 (86.31, 100.00)	0.00	-†
LAD *V*_20_ (%)	95.27 (78.27, 100.00)	0.00	-†
LAD *V*_30_ (%)	84.51 (68.69, 99.83)	0.00	-†
LAD *V_4_*_0_ (%)	59.53 (48.91, 75.84)	0.00	-†
Right coronary artery (RCA)			
RCA *D*_mean_ (Gy)	4.14 (3.20, 5.44)	12.38 (9.17, 15.23)	<0.001
RCA *D*_max_ (Gy)	5.61 (4.26, 8.30)	18.68 (14.43, 24.42)	<0.001
RCA *V*_5_ (%)	17.95 (0.00, 66.71)	100.00 (94.99, 100.00)	<0.001
RCA *V*_10_ (%)	0.00	67.06 (37.06, 97.45)	-†

Data are presented as Median (25th percentile, 75th percentile). MHD, Mean Heart Dose. ^†^*P* value not calculated because the statistical difference is self-evident due to absolute zero vs. positive range separation.

Left-sided Biased Substructures: Mean heart dose (MHD) and global mid-to-high dose volumes (Heart *V*_10_–*V*_30_) were significantly higher in left-sided patients (all *P* < 0.001). Left-sided patients sustained absolute elevations in left ventricular (LV) *D*_mean_ (9.49 vs. 2.15 Gy), LV *D*_max_ (50.39 vs. 7.48 Gy), and all LV volumetric metrics (*V*_5_–*V*_40_) (all *P* < 0.001). Similarly, all dosimetric parameters for the left anterior descending artery (LAD) comprehensively peaked during left-sided treatment (all *P* < 0.001).

Right-sided Biased Substructures: Conversely, right-sided treatment led to pronounced dose escalations in right-sided sub-volumes (all *P* < 0.001), including SAN *D*_mean_ (8.22 vs. 3.13 Gy), SAN *D*_max_ (13.16 vs. 4.85 Gy), and SAN *V*_5_ (100% vs. 0%). All right coronary artery (RCA) indices also comprehensively exceeded those of the left-sided group (all *P* < 0.001). The central-right atrioventricular node (AVN) sustained higher doses regarding *D*_max_ (6.51 vs. 4.30 Gy, *P* = 0.009) and V_5_ (19.27% vs. 0%, *P* = 0.012).

Right Ventricle (RV) Exposure: Notably, the right ventricle (RV) received significantly higher doses during left-sided treatment *D*_mean_: 9.79 Gy; *D*_max_: 47.90 Gy) than right-sided treatment (*D*_mean_: 5.12 Gy; *D*_max_: 16.65 Gy) (both *P* < 0.001).

### 3.2 Follow-up of ECG abnormalities and event-free survival

The median follow-up was 12 months post-radiotherapy. Kaplan-Meier survival analysis (**Figure 1A**) demonstrated that left-sided patients experienced a significantly shorter early ECG abnormality-free survival time than right-sided patients (median: 7.0 vs. 12.0 months; Log-rank *P* = 0.038). At 3-, 6-, and 12-months post-RT, the cumulative incidence rates of ECG abnormalities were 5.9%, 39.4%, and 70.5% for left-sided patients, versus 10.8%, 20.1%, and 50.9% for right-sided patients, respectively. Cumulative hazard curves (**Figure 1B**) confirmed a persistently higher dynamic risk in the left-sided group, with a progressive divergence expanding markedly at approximately 6 months post-radiotherapy.

**Figure 1:**
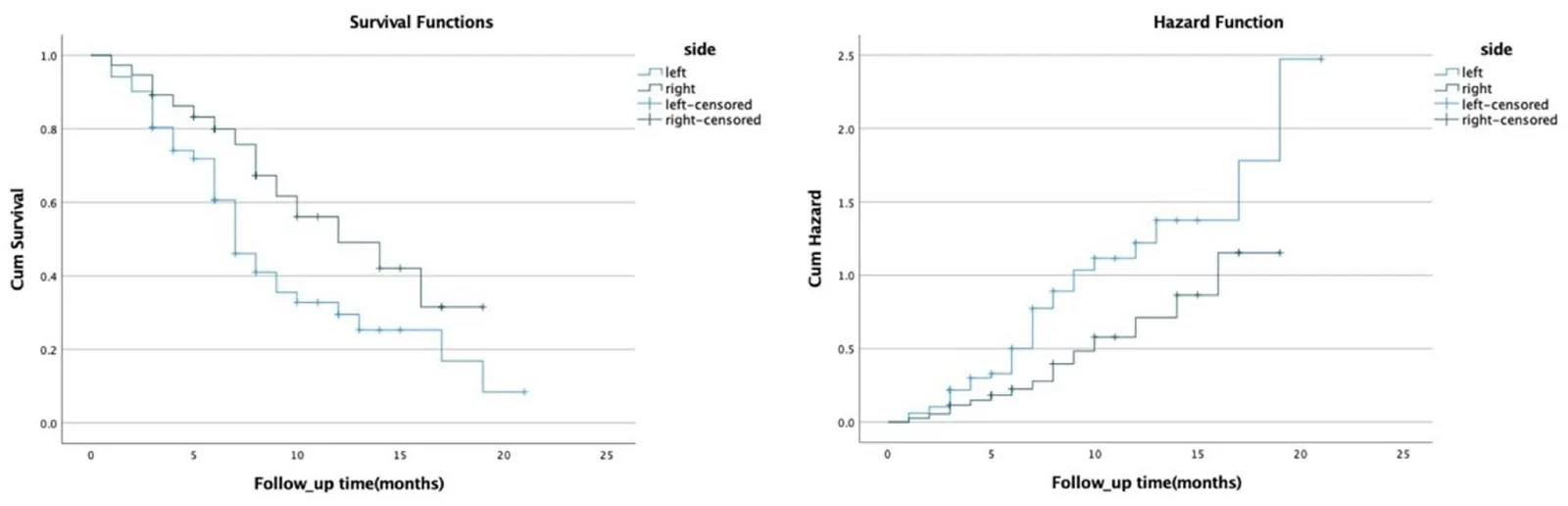
**(A)** Kaplan-Meier curves for early ECG abnormality-free survival following radiotherapy in left-sided versus right-sided breast cancer patients. **(B)** Cumulative hazard function curves demonstrating a persistently higher risk of ECG abnormality surrogates in left-sided patients.

### 3.3 Spatial heterogeneity in the distribution of ECG abnormality phenotypes by laterality

Post-radiotherapy, 60 of the 91 patients developed new-onset early ECG abnormality surrogates (overall incidence: 65.9%), comprising 44 left-sided (incidence: 81.5%) and 16 right-sided cases (incidence: 43.2%). Stratification of these 60 patients revealed highly significant laterality-specific selectivity in phenotype distribution (rhythm vs. repolarization; Pearson’s χ² = 4.602, *P* = 0.032; Fisher’s exact test, two-sided *P* = 0.041). As detailed in **Table 2**, cardiac injury in left-sided patients was predominantly characterized by repolarization abnormalities (ST-T changes and QTc prolongation), accounting for a majority of 68.2% (30/44). Conversely, right-sided patients mainly presented with new-onset rhythm abnormalities (sinus arrhythmia, tachycardia, or premature beats), reaching 62.5% (10/16).

**Table 2:** Cross-distribution of anatomical laterality and electrophysiological abnormality types in patients with new-onset ECG abnormalities.

Laterality	Rhythm abnormality (n, %)	Repolarization abnormality (n, %)	Total
Left-sided	14 (31.8)	30 (68.2)	44
Right sided	10 (62.5)	6 (37.5)	16
Total	24 (40.0)	36 (60.0)	60

Pearson’s *χ²* = 4.602, *df* = 1, *P* = 0.032 (Fisher’s exact test, two-tailed *P* = 0.041).

### 3.4 Left ventricular dose as a driver of repolarization abnormalities within the left-sided subgroup

To elucidate the specific physical drivers of the high repolarization abnormalities incidence on the left-sided patients, the 44 left-sided patients with early ECG abnormalities were stratified into a repolarization (n=30) and rhythm (n=14) subgroups for detailed DVH parameters comparison (**Table 3**).

**Table 3:** Dosimetric analysis of cardiac structures between the repolarization and rhythm groups in left-sided breast cancer patients with new-onset ECG abnormalities.

Parameter	Repolarization abnormality (n=30)	Rhythm abnormality (n=14)	*P* value
Whole heart			
MHD (Gy)	7.96 (7.11, 9.99)	7.09 (6.24, 7.98)	0.02
heart *D*_max_ (Gy)	52.97 (51.01, 55.35)	50.58 (47.35, 52.59)	0.02
heart *V*_5_ (%)	38.81 (31.56, 50.47)	34.97 (28.03, 40.68)	0.22
heart *V*_10_ (%)	20.41 (16.03, 26.66)	15.55 (12.42, 20.79)	0.03
heart *V*_20_ (%)	11.79 (9.13, 14.96)	9.27 (6.23, 10.94)	0.009
heart *V*_30_ (%)	7.13 (5.53, 8.40)	4.31 (3.34, 6.12)	0.001
heart *V_4_*_0_ (%)	2.63 (1.46, 4.11)	1.16 (0.48, 2.81)	0.01
Sinoatrial node (SAN)			
SAN *D*_mean_ (Gy)	3.09 (2.36, 4.38)	3.32 (2.47, 4.42)	0.64
SAN *D*_max_ (Gy)	4.64 (3.66, 5.64)	4.64 (3.63, 5.90)	0.91
SAN *V*_5_ (%)	0.00 (0.00, 7.48)	0.00 (0.00, 44.22)	0.60
Atrioventricular node (AVN)			
AVN *D*_mean_ (Gy)	3.20 (2.29, 4.38)	2.69 (2.30, 3.79)	0.51
AVN *D*_max_ (Gy)	5.61 (2.94, 6.66)	3.67 (3.29, 5.54)	0.36
AVN *V*_5_ (%)	4.55 (0.00, 22.74)	0.00 (0.00, 6.87)	0.13
Left ventricle (LV)			
LV *D*_mean_ (Gy)	10.10 (8.97, 11.01)	7.97 (7.32, 9.02)	0.003
LV *D*_max_ (Gy)	51.23 (47.29, 53.22)	48.37 (45.89, 51.64)	0.12
LV *V_5_* (%)	39.24 (34.87, 49.58)	33.57 (29.16, 37.25)	0.005
LV *V*_10_ (%)	26.19 (22.37, 30.19)	19.32 (18.16, 26.12)	0.003
LV *V*_20_ (%)	17.65 (14.03, 20.24)	12.39 (9.17, 16.15)	0.004
LV *V*_30_ (%)	10.05 (7.40, 12.33)	6.48 (4.21, 8.83)	0.004
LV *V_4_*_0_ (%)	3.85 (1.15, 5.98)	1.63 (0.73, 3.64)	0.08
Right ventricle (RV)			
RV *D*_max_ (Gy)	49.07 (46.21, 51.78)	44.1 (37.18, 49.71)	0.03
RV *D*_mean_ (Gy)	10.98 (7.94, 13.15)	8.45 (6.17, 11.10)	0.05
RV *V*_5_ (%)	53.74 (47.04, 63.11)	49.98 (37.76, 64.06)	0.48
RV *V*_10_ (%)	30.09 (22.44, 38.76)	21.67 (11.94, 34.51)	0.08
RV *V*_20_ (%)	18.40 (9.42, 24.45)	11.47 (3.01, 17.41)	0.03
RV *V*_30_ (%)	9.52 (3.97, 13.58)	5.23 (0.36, 7.86)	0.01
Left anterior descending artery (LAD)			
LAD *D*_max_ (Gy)	51.61 (49.61, 54.18)	50.99 (46.82, 52.54)	0.23
LAD *D*_mean_ (Gy)	40.04 (37.71, 44.67)	39.70 (32.22, 44.13)	0.48
LAD *V*_5_ (%)	100.00 (100.00, 100.00)	100.00 (96.87, 100.00)	0.39
LAD *V*_10_ (%)	99.92 (87.21, 100.00)	99.53 (82.80, 100.00)	0.79
LAD *V*_20_ (%)	97.08 (82.97, 100.00)	97.54 (74.02, 100.00)	0.79
LAD *V*_30_ (%)	89.94 (72.23, 99.93)	88.40 (66.46, 96.89)	0.61
LAD *V_4_*_0_ (%)	63.68 (58.32, 82.25)	54.01 (48.03, 78.39)	0.19
Right coronary artery (RCA)			
RCA *D*_max_ (Gy)	5.49 (4.19, 8.65)	5.22 (4.20, 8.80)	0.79
RCA *D*_mean_ (Gy)	3.74 (2.85, 5.45)	3.93 (3.30, 5.45)	0.88
RCA *V*_5_ (%)	14.15 (0.00, 58.65)	1.27 (0.00, 67.80)	0.49

Due to space limitations, some volumetric parameters for SAN, AVN, and RCA that showed no significant inter-group differences (*P* > 0.10) are omitted from the table. MHD, Mean Heart Dose.

Left ventricular (LV) physical dose exposure metrics were significantly higher in the repolarization subgroup. Specifically, LV *D*_mean_ was markedly elevated (10.10 Gy vs. 7.97 Gy, *P* = 0.003), with significant statistical increases in LV *V*_5_ (*P* = 0.005), LV *V*_10_ (*P* = 0.003), LV *V*_20_ (*P* = 0.004), and LV *V*_30_ (*P* = 0.004). Whole heart dosimetric indices, including MHD (7.96 Gy vs. 7.09 Gy, *P* = 0.020), Heart *D*_max_ (52.97 Gy vs. 50.58 Gy, *P* = 0.020), and mid-to-high dose volumes (Heart *V*_10_- *V*_40_), were similarly elevated in the repolarization subgroup (all *P* < 0.05). Notably, significantly higher high-dose exposure to the right ventricle (RV *D*_max_: 49.07 vs. 44.10 Gy, *P* = 0.030; RV V_20_–V_80_, all *P* < 0.05) and higher RV *D*_mean_ (10.98 vs. 8.45 Gy, *P* = 0.050) were also documented in the repolarization subgroup, structurally reflecting the anterior anatomical proximity and field overlap under free-breathing extended regional fields.

Conversely, the very high-dose volume integrity of LV V_40_ (3.85% vs. 1.63%, *P* = 0.080) and all dosimetric parameters for conduction structures (SAN, AVN) or main coronary artery tracks (LAD, RCA) showed no statistical inter-group differences (all *P* > 0.05).

### 3.5 ROC curve analysis and determination of dosimetric thresholds

Receiver operating characteristic (ROC) curve analysis (**Figure 2**; **Table 4**) evaluated the capacity of global and region-specific dosimetric metrics to predict new-onset early repolarization abnormality surrogates in left-sided patients.

**Figure 2:**
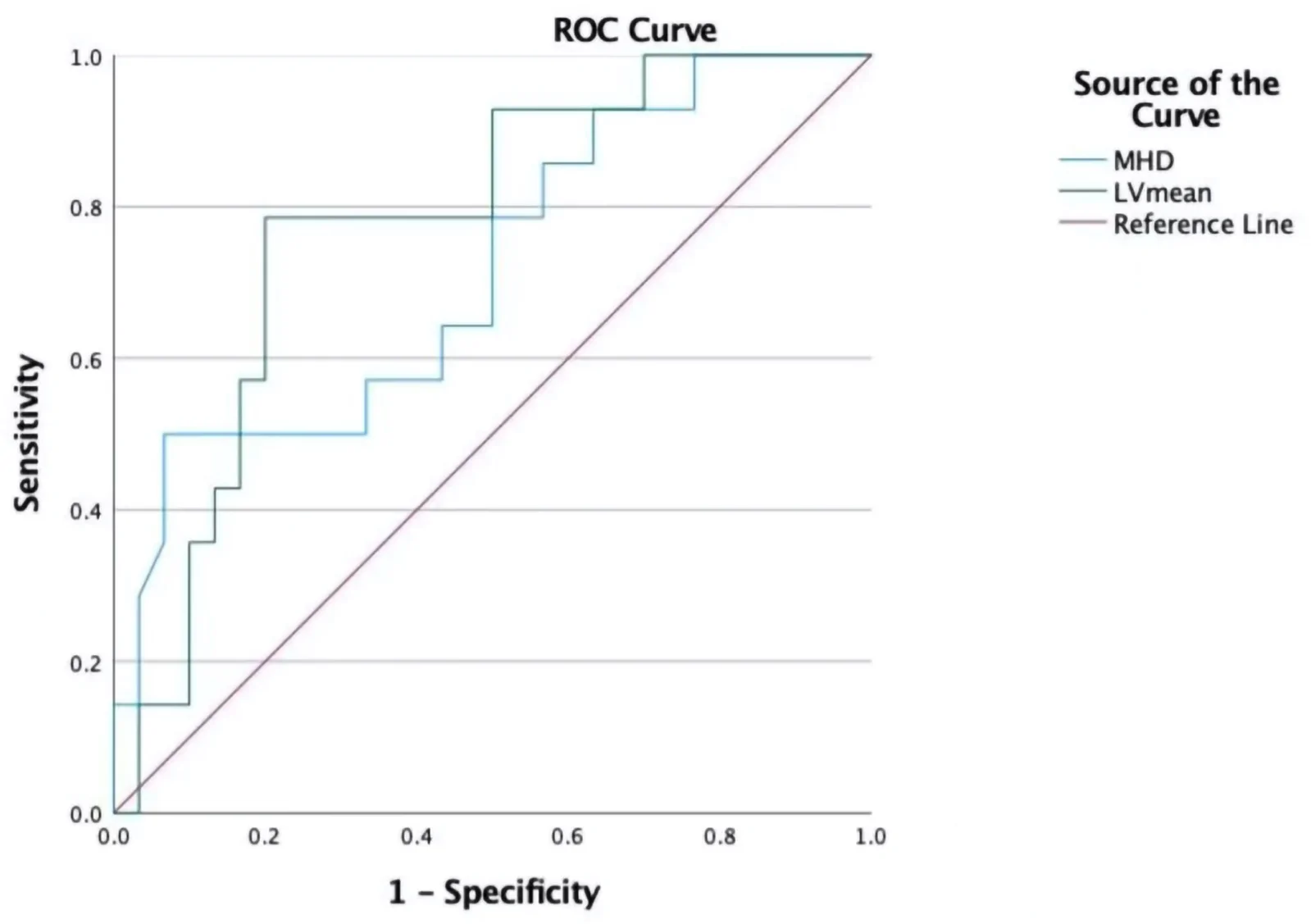
Receiver operating characteristic (ROC) curves of MHD and LV *D*_mean_ for early post-RT repolarization alterations in left-sided breast cancer patients.

**Table 4:** ROC curve analysis parameters of MHD and LV *D*_mean_ for predicting post-radiotherapy repolarization abnormalities.

Parameter	MHD	LV *D*_mean_
AUC	0.715	0.776
95% CI	0.55 - 0.88	0.63 - 0.92
*P* value	0.020	0.003
Split-point (Gy)	8.10	8.75
Sensitivity (%)	43.3	80.0
Specificity (%)	85.7	78.6

ROC, receiver operating characteristic; MHD, mean heart dose; LV *D*_mean_, left ventricular mean dose; CI, confidence interval.

The area under the curve (AUC) for LV *D*_mean_ (AUC = 0.776, 95% CI: 0.633–0.920, *P* = 0.003) mathematically outperformed that of the traditional global metric MHD (AUC = 0.715, 95% CI: 0.550–0.880, *P* = 0.023). Utilizing maximal selected rank statistics via the “maxstat” package in R software to optimize association with time-to-event outcomes, the volume-specific, toxicity-associated cutoff values were determined. The statistical split-point for MHD was 8.10 Gy (sensitivity: 43.3%, specificity: 85.7%), whereas the split-point for sub-volume LV *D*_mean_ was 8.75 Gy (sensitivity: 80.0%, specificity: 78.6%).

These findings indicate that within this specific historical cohort, the left ventricular-specific mean dose achieves enhanced mathematical association with early, transient post-RT electrophysiological alterations.

## 4 Discussion

This study systematically quantified the correspondence between anatomical laterality and physical dose distribution among various cardiac substructures under historical free-breathing IMRT for breast cancer. The clinical and dosimetric evidence strongly supports a laterality-specific “dual-mechanism model” descriptive of early, acute/subacute post-radiotherapy (post-RT) electrophysiological alterations, rather than permanent myocardial damage. Specifically, early post-RT ECG modifications induced by left-sided breast cancer are primarily mediated by extensive physical exposure to the anterior cardiac substructures, structurally involving both the diffuse LV myocardium and the anterior wall of the RV, manifesting as early, acute/subacute repolarization alterations. Conversely, acute physiological manifestations induced by right-sided treatments are characterized by rhythm stress from focal exposure of right-sided conduction substructures—principally the SAN—which are precisely trapped within the high-dose penumbra of right-sided beam arrangements.

### 4.1 Electrophysiological vulnerability of conduction system

In the present cohort, patients with right-sided breast cancer displayed an ECG variation spectrum fundamentally distinct from that of left-sided patients. Although the overall incidence of post-RT alterations was relatively lower in the right-sided group due to the general levo-position of the heart (43.2% vs. 81.5%), rhythm alterations were overwhelmingly dominant among right-sided patients who developed new-onset ECG modifications (62.5% vs. 31.8% in the left-sided group).

This phenotypic presentation aligns with the physical dose topography of right-sided treatments. During multi-field IMRT for right-sided breast cancer, accommodating the chest wall and internal mammary lymph node targets inevitably requires beam arrangements that directly expose the SAN to localized elevated doses. Our DVH statistics confirmed that right-sided patients sustained a median SAN *D*_mean_ of 8.22 Gy and SAN V_5_ of 100%, whereas these parameters were virtually zero in the left-sided group. Concurrently, the maximum dose and V_5_ of AVN were also significantly elevated in right-sided plans (*D*_max_: 6.51 vs. 4.30 Gy, *P* = 0.009; V_5_: 19.27% vs. 0%, *P* = 0.012), confirming the direct physical coverage of right-sided pathways by right-sided beam trajectories.

Basic cellular electrophysiology indicates that pacemaker cells exhibit non-linear susceptibility to ionizing radiation. Radiation-induced reactive oxygen species (ROS) can compromise the microvascular endothelial barrier and disrupt the expression and opening kinetics of hyperpolarization-activated cyclic nucleotide-gated channels (HCN4, mediating the *I*_f_ current) and L-type calcium channels (*I*_Ca,L_)) on the membranes of SAN pacemaker cells (16). This ion channel and electrical remodeling can impair the automaticity and conduction stability of the SAN, triggering acute sinus rhythm disturbances (17). Within the right-sided subgroup, dosimetric parameters of the SAN did not differ significantly between the rhythm and repolarization cohorts due to a universal “high-dose saturation” status (SAN V_5_ > 80%), which masked linear dose-response relationships. Nevertheless, damage to the pacemaker complex may operate via a non-linear “trigger effect” rather than a purely linear model. High-precision electrophysiological tracking has confirmed that even low-to-moderate scatter doses flanking the right-sided fields can disrupt nodal architecture (11), and localized hotspots in conduction substructures trigger incident tachyarrhythmias far more deterministically than global whole-heart metrics (18).

### 4.2 Left-sided breast cancer and repolarization surrogates

In left-sided breast cancer IMRT under historical free-breathing conditions, the LV and LAD sustained substantial physical dose exposure owing to their unavoidable proximity to the left chest wall and internal mammary targets. Follow-up observations revealed that early electrophysiological modifications in left-sided patients typically presented as acute/subacute repolarization alterations (68.2%), which correlated with a significantly compromised event-free survival time (median 7.0 vs. 12.0 months, *P* = 0.038).

Subgroup analysis nested within the left-sided cohort demonstrated that all LV dosimetric parameters (LV *D*_mean_, LV *V*_5_–*V*_30_) in the repolarization alteration subgroup were significantly higher than those in the rhythm alteration group. This finding high light that the cumulative radiation volume sustained by the LV myocardium, particularly large-volume exposure to low-to-moderate dose zones, serves as the critical initiator inducing early, transient ST-T changes and QTc prolongation.

The underlying pathophysiology is primarily tied to myocardial microvascular stress induced by radiation. Following extensive low-to-moderate dose irradiation of the LV (the median LV V_5_ reached 37.96% in left-sided cases), radiation triggers apoptosis of microvascular endothelial cells, luminal occlusion, and localized microcirculatory hypoxia. Hypoxia signals activate the transforming growth factor-beta (TGF-β) pathway, prompting proliferation of fibroblasts and their trans-differentiation into myofibroblasts, leading to interstitial fibrosis (19). This fibrosis alters local mechanical compliance and hinders gap junctional conduction between cardiomyocytes (e.g., via the downregulation of Connexin 43), leading to heterogeneity in action potential duration prolongation across different myocardial layers, capturing as transient ST-T changes and QTc prolongation on surface ECGs (20).

Interestingly, the high-dose volume parameter LV *V*_40_ did not achieve statistical significance between the two subgroups (P = 0.080), demonstrating that strict treatment planning constraints successfully restricted the absolute high-dose volumes (> 40 Gy) of the LV. Conversely, large-volume infiltration of the low-to-moderate dose regions (*V*_5_–*V*_80_), which was historically accepted to secure comprehensive PTV and IMN coverage, acts as the primary physical driver accelerating early subclinical myocardial repolarization alterations. This driven by low-dose volumes is corroborated by prospective ^99m^Tc--sestamibi SPECT tracking, demonstrating that left-sided patients exhibit localized myocardial perfusion defects mapping directly to the LV segments enveloped by the *V*_5_–*V*_80_ low-dose bath (5, 21). Similarly, global longitudinal strain rate reductions captured via cardiac MRI further validate that large-volume low-dose myocardial infiltration serves as the fundamental instigator of early subclinical LV strain variations (22).

### 4.3 Statistical interpretation of splitting metrics and real-world technical transition

Historically, the radiation oncology community widely adopted the global MHD as a universal metric to predict overall cardiotoxicity risks (4). However, our ROC curve analysis indicates that for identifying new-onset early repolarization alterations in left-sided patients, the sub-volume metric LV *D*_mean_ exhibited a mathematically superior area under the curve (AUC = 0.776) compared with the traditional global MHD (AUC = 0.715). Crucially, at these mathematical split-points, the diagnostic sensitivity of MHD was poor at only 43.3%, whereas LV *D*_mean_ enhanced the mathematical association sensitivity to 80.0%. This indicates that because the global MHD incorporates non-irradiated volumes into its average calculation, it can mathematically dilute the intensive physical radiation exposure sustained by the anterior left ventricular and adjacent anterior right ventricular sub-volumes (23). This diagnostic distinction has been recognized in recent clinical registries, indicating that relying solely on global MHD systematically underestimates localized exposure profiles under comprehensive regional fields.

We must strongly emphasize that the mathematically derived statistical split-points obtained in this study (MHD of 8.10 Gy and LV *D*_mean_ of 8.75 Gy) are purely population-specific descriptors characterizing this specific historical high-exposure cohort and must NOT be interpreted as universal clinical safe constraints. Furthermore, the 81.5% incidence of early ECG modifications observed in our left-sided cases represents acute, subacute, and potentially transient physiological responses to radiation-induced localized inflammation, rather than permanent myocardial damage or clinical major adverse cardiac events (MACE).

Reaffirming our commitment to the strict ALARA principles, our department has successfully implemented the advanced Swedish C-RAD optical surface management system (OSMS) to fully establish the VMAT combined with Deep Inspiration Breath-Hold (DIBH) workflow in our clinical routine. In our modern clinical practice, this advanced VMAT+DIBH setup succeeds in substantially optimizing cardiac sparing compared to historical baselines, effectively minimizing the MHD for both routine hypofractionated plans and complex, high-risk conventional fractionation protocols that mandate extensive internal mammary node irradiation (IMNI). This technological transition achieves enhanced physical protection for both the anterior ventricular chambers and right-sided conduction substructures. Consequently, evaluating this past free-breathing cohort serves as a highly valuable real-world retrospective benchmark that underscores the strict clinical necessity and urgency of transitioning to modern VMAT+DIBH technologies at our institution to minimize sub-volume low-dose paths.

### 4.4 Limitations

The present study has several clinical limitations. First, it relies on a single-center, retrospective design with a historical cohort under free-breathing conditions, capturing early, acute post-RT electrophysiological variations on ECGs rather than long-term hard clinical endpoints of RIHD, such as permanent ischemic events or chronic heart failure over an extended period. Second, constrained by the sample size of specific sub-volumes, certain subgroup analyses may have suffered from statistical power attenuation, requiring caution during validation. Furthermore, serum biomarkers such as hs-cTnI or BNP/NT-proBNP were not incorporated for cross-validation in this past timeframe. Future multi-center, prospective validation cohorts integrating myocardial speckle tracking echocardiography and longitudinal serological tracking are warranted to evaluate the long-term clinical relevance of these early physiological surrogates.

## 5 Conclusion

Early post-radiotherapy ECG alterations follow a laterality-specific dual-mechanism model. Left-sided modifications manifest primarily as acute repolarization surrogates driven by diffuse physical exposure to anterior cardiac structures (the LV and anterior RV wall). Conversely, right-sided modifications present as rhythm-based acute stress responses from focal exposure of right-sided conduction substructures, particularly the sinoatrial node. Advanced techniques like VMAT combined with DIBH are strongly advocated to minimize cardiac sub-volume exposure.

## Data Availability

The raw data supporting the conclusions of this article will be made available by the authors, without undue reservation.

## References

[1] Kunkler IH Russell NS Anderson N Sainsbury R Dixon JM Cameron D . Ten-year survival after postmastectomy chest-wall irradiation in breast cancer. N Engl J Med. (2025) 393:1771–83. doi: 10.1056/nejmoa2412225 41191939 PMC7618363

[2] Bergom C Bradley JA Ng AK Samson P Robinson C Lopez-Mattei J . Past, present, and future of radiation-induced cardiotoxicity: Refinements in targeting, surveillance, and risk stratification. JACC CardioOncol. (2021) 3:343–59. doi: 10.1016/j.jaccao.2021.06.007 34604796 PMC8463722

[3] Darby SC Ewertz M McGale P Bennet AM Blom-Goldman U Brønnum D . Risk of ischemic heart disease in women after radiotherapy for breast cancer. N Engl J Med. (2013) 368:987–98. doi: 10.1056/nejmoa1209825 23484825

[4] Bowen Jones S Marchant T Saunderson C McWilliam A Banfill K . Moving beyond mean heart dose: The importance of cardiac substructures in radiation therapy toxicity. J Med Imaging Radiat Oncol. (2024) 68:974–86. doi: 10.1111/1754-9485.13737 39228181 PMC11686456

[5] Zagar TM Kaidar-Person O Tang X Jones EE Matney J Das SK . Utility of deep inspiration breath hold for left-sided breast radiation therapy in preventing early cardiac perfusion defects: A prospective study. Int J Radiat Oncol Biol Phys. (2017) 97:903–9. doi: 10.1016/j.ijrobp.2016.12.017 28333011

[6] Lyon AR López-Fernández T Couch LS Asteggiano R Aznar MC Bergler-Klein J . 2022 ESC guidelines on cardio-oncology developed in collaboration with the European Hematology Association (EHA), the European Society for Therapeutic Radiology and Oncology (ESTRO) and the International Cardio-Oncology Society (IC-OS). Eur Heart J. (2022) 43:4229–361. doi: 10.1093/ehjci/jeac106 36017568

[7] Skyttä T Tuohinen S Boman E Virtanen V Raatikainen P Kellokumpu-Lehtinen PL . Troponin T-release associates with cardiac radiation doses during adjuvant left-sided breast cancer radiotherapy. Radiat Oncol. (2015) 10. doi: 10.1186/s13014-015-0436-2 PMC4496940 26159409

[8] Li X Wu Y Wang Q Li B Wang J . Radiation-induced cardiac substructure damage and dose constraints: A review. Radiat Oncol. (2025) 20:94. doi: 10.1186/s13014-025-02668-x 40474260 PMC12139131

[9] Pouvreau P Taleb I Fontaine A Edouard L Gibson N Yaouanq M . Heart is a heavy burden: Cardiac toxicity in radiation oncology. Support Care Cancer. (2024) 32:769. doi: 10.1007/s00520-024-08949-7 39495349

[10] Errahmani MY Locquet M Broggio D Spoor D Jimenez G Camilleri J . Supraventricular cardiac conduction system exposure in breast cancer patients treated with radiotherapy and association with heart and cardiac chambers doses. Clin Transl Radiat Oncol. (2023) 38:62–70. doi: 10.1016/j.ctro.2022.10.015 36388244 PMC9646649

[11] Errahmani MY Locquet M Spoor D Jimenez G Camilleri J Bernier MO . Association between cardiac radiation exposure and the risk of arrhythmia in breast cancer patients treated with radiotherapy: A case-control study. Front Oncol. (2022) 12. doi: 10.1016/j.acvdsp.2022.10.317 35860581 PMC9289188

[12] Walker V Lairez O Fondard O Pathak A Pinel B Chevelle C . Early detection of subclinical left ventricular dysfunction after breast cancer radiation therapy using speckle-tracking echocardiography: Association between cardiac exposure and longitudinal strain reduction (BACCARAT study). Radiat Oncol. (2019) 14:204. doi: 10.1186/s13014-019-1408-8 31727075 PMC6854785

[13] Zhang Y Han X Chi Z Zhao Z Wang F Liu D . Radiation exposure and clinical validation of autosegmentation models for the supraventricular cardiac conduction system in breast cancer radiotherapy: An institutional perspective. Front Oncol. (2026) 16. doi: 10.3389/fonc.2026.1734696 41695359 PMC12893944

[14] Loap P Servois V Dhonneur G Kirov K Fourquet A Kirova Y . A radiation therapy contouring atlas for cardiac conduction node delineation. Pract Radiat Oncol. (2021) 11:e434–e7. doi: 10.1016/j.prro.2021.02.002 33561549

[15] Loap P De Marzi L Kirov K Servois V Fourquet A Khoubeyb A . Development of simplified auto-segmentable functional cardiac atlas. Pract Radiat Oncol. (2022) 12:533–8. doi: 10.1016/j.prro.2022.02.004 35192938

[16] Zhang DM Navara R Yin T Szymanski J Goldsztejn U Kenkel C . Cardiac radiotherapy induces electrical conduction reprogramming in the absence of transmural fibrosis. Nat Commun. (2021) 12:5558. doi: 10.1038/s41467-021-25730-0 34561429 PMC8463558

[17] Thorolfsdottir RB Sveinbjornsson G Aegisdottir HM Benonisdottir S Stefansdottir L Ivarsdottir EV . Genetic insight into sick sinus syndrome. Eur Heart J. (2021) 42:1959–71. doi: 10.1093/eurheartj/ehaa1108 33580673 PMC8140484

[18] Keane FK Neilan TG Jimenez RB . Elucidating the signal from the noise: Mapping cardiac conduction events to cardiac substructure radiation exposure. JACC CardioOncol. (2024) 6:946–8. doi: 10.1016/j.jaccao.2024.10.007 39801636 PMC11711799

[19] Liu LK Ouyang W Zhao X Su Sh F Yang Y Ding WJ . Pathogenesis and prevention of radiation-induced myocardial fibrosis. Asian Pac J Cancer Prev. (2017) 18:583–7. doi: 10.22034/APJCP.2017.18.3.583 PMC5464468 28440606

[20] Taunk NK Haffty BG Kostis JB Goyal S . Radiation-induced heart disease: Pathologic abnormalities and putative mechanisms. Front Oncol. (2015) 5. doi: 10.3389/fonc.2015.00039 25741474 PMC4332338

[21] Omidi A Weiss E Trankle CR Rosu-Bubulac M Wilson JS . Quantitative assessment of radiotherapy-induced myocardial damage using MRI: A systematic review. Cardiooncology. (2023) 9:24. doi: 10.1186/s40959-023-00175-0 37202766 PMC10193692

[22] Lamy J Spoor DS Langendijk JA Vliegenthart R Eraso A Ventura M . Cardiac MRI-based subclinical cardiac dysfunction during 2 years after breast cancer irradiation: The MEDIRAD EARLY-HEART study. Radiol Cardiothorac Imaging. (2025) 7:e240231. doi: 10.1148/ryct.240231 40178396

[23] Nuruddeen MG A Karim NK A L Appalanaido GK Mohd Zin MH Sayuti KA Mad Naser MN . Cardiac substructure dose distributions in node-positive and node-negative breast cancer patients undergoing 3D-CRT: Comparing the predictive accuracy of mean heart dose and mean left ventricular dose. Radiat Oncol. (2025) 20:65. doi: 10.1186/s13014-025-02607-w 40301974 PMC12039179

