# Supplementary material for: Beyond mean heart dose: differentiated electrocardiographic manifestations and dosimetric mechanisms of cardiac injury in left-sided versus right-sided breast cancer after intensity-modulated radiotherapy
Source: Front Oncol. 2026 Sep 16;16:1901038. doi: 10.3389/fonc.2026.1901038 (PMC13623708; doi:10.3389/fonc.2026.1901038)
Supplement: Supplementary file 1 [file DataSheet1.docx]

Supplementary Table 1. Baseline clinical and comorbidity characteristics of the study population.

| Characteristic | Left-sided (n=54) | Right-sided (n=37) | *χ2*/*Z* value | *P* value |
| --- | --- | --- | --- | --- |
| Age (years), M (P25, P75) | 53 (43, 60) | 51 (43.5, 59) | 0.36 | 0.76 |
| Chemotherapy, *n (%)* |  |  | 0.31 | 0.45 |
| Yes | 42 (77.80%) | 28 (75.70%) |  |  |
| No | 12 (22.20%) | 9 (24.30%) |  |  |
| Targeted therapy, *n (%)* |  |  | 0.60 | 0.22 |
| Yes | 20 (37.00%) | 10 (27.00%) |  |  |
| No | 33 (63.00%) | 27 (73.00%) |  |  |
| Endocrine therapy, *n (%)* |  |  | 0.80 | 0.11 |
| Yes | 33 (61.10%) | 18 (48.60%) |  |  |
| No | 21 (38.90%) | 19 (51.40%) |  |  |
| Hypertension, *n (%)* |  |  | 0.09 | 0.83 |
| Yes | 12 (22.20%) | 8 (21.60%) |  |  |
| No | 42 (77.80%) | 29 (78.40%) |  |  |
| Dyslipidemia, *n (%)* |  |  | 0.06 | 0.90 |
| Yes | 20 (37.00%) | 14 (37.80%) |  |  |
| No | 33 (63.00%) | 23 (62.20%) |  |  |
| Diabetes mellitus, *n (%)* |  |  | 0.17 | 0.58 |
| Yes | 6 (11.10%) | 3 (8.10%) |  |  |
| No | 48 (88.90%) | 34 (91.90%) |  |  |

Supplementary table S2. Target volume classification and localized boost specifications for left-sided and right-sided breast cancer patients.

| Classification | Target | Left(n=54) | Right(n=37) |
| --- | --- | --- | --- |
| Target Volume Classification | ptvcw+ptvsc+ptvim | 28(51.9%) | 17(45.9%) |
|  | ptvcw+ptvsc+ptvim+ptvax | 3(5.6%) | 1(2.7%) |
|  | ptvcw+ptvsc | 6(11.1%) | 1(2.7%) |
|  | ptv | 9(16.7%) | 9(24.3%) |
|  | ptv+ptvsc | 5(9.3%) | -- |
|  | ptv+ptvsc+ptvim | 3(5.6%) | 8(21.6%) |
|  | ptvcw+ptvsc+ptvax | -- | 1(2.7%) |
| Localized Boost Specification | ptvboost | 17（31.5%） | 17（45.9%） |
|  | ptvimnd | 3（5.6%） | 2（5.4%） |
|  | ptvscnd | 3（5.6%） | 2（5.4%） |
|  | ptvimnd+ ptvscnd | 4(7.4%) | -- |
|  | no boost | 27（50%） | 16（43.2%） |

Note：ptvcw = PTV_Chest Wall；ptv = PTV_Breast；ptvsc = PTV_Supraclavicular；ptvscnd = PTV_Supraclavicular node；ptvim = PTV_IMN；ptvimnd = PTV_IMN node；ptvax = PTV_Axilla；Ptvboost = PTV_Tumor Bed Boost

|  |  |
| --- | --- |
|  |  |

Supplementary figure S1. Target volume classification and localized boost specifications for left-sided and right-sided breast cancer patients.

**(A)** Axial dose isodose line image demonstrating that due to strict anatomical proximity, the anterior wall of the right ventricle (RV) and the left anterior descending artery (LAD) are inevitably enveloped by the high-dose gradient flanking the left internal mammary node target volume (PTVim).

**(B)** Beam arrangement layout illustrating multi-field intensity-modulated radiotherapy (IMRT) trajectories intersecting through the anterior cardiac structures to achieve prescription targets.

Supplementary Figure S2. Representative treatment planning specifications and dosimetric distributions for a left-sided breast cancer patient receiving comprehensive regional and internal mammary node irradiation.
